# One-Step Regeneration of Hairy Roots to Induce High Tanshinone Plants in *Salvia miltiorrhiza*

**DOI:** 10.3389/fpls.2022.913985

**Published:** 2022-05-20

**Authors:** Yuekai Su, Caicai Lin, Jin Zhang, Bei Hu, Jie Wang, Jingyu Li, Shiqi Wang, Ruihao Liu, Xia Li, Zhenqiao Song, Jianhua Wang

**Affiliations:** ^1^State Key Laboratory of Crop Biology, Shandong Key Laboratory of Crop Biology, College of Agronomy, Shandong Agricultural University, Tai’an, China; ^2^Taishan Academy of Forestry Sciences, Tai’an, China

**Keywords:** *Salvia miltiorrhiza*, genetic transformation, *Agrobacterium rhizogenes*, hairy root, regeneration, tanshinones

## Abstract

*Salvia miltiorrhiza* is a traditional Chinese medicinal plant of Labiatae, which has been widely utilized to treat a variety of cardiovascular and cerebrovascular diseases. However, due to the long growth cycle, low content of active ingredients, and serious quality deterioration of *S. miltiorrhiza*, the use of biotechnology to improve *S. miltiorrhiza* to meet the growing demand for clinical applications has become a research hotspot. In this study, a novel one-step hairy root regeneration method was developed, which could rapidly obtain hairy roots and regenerated plants with high tanshinone content. By optimizing the parameters of *Agrobacterium rhizogenes* transformation in *S. miltiorrhiza*, it was finally established that the explants were infected in *Ar.qual* (OD_600_ = 0.6) for 10 min, co-cultured for 3 days, and then screened on the screening medium containing 7.5 mg/l hygromycin, the maximum transformation frequency can reach 73.85%. GFP and PCR detection yielded a total of 9 positive transgenic hairy root lines and 11 positive transgenic regenerated plants. *SmGGPPS1* was successfully overexpressed in positive transgenic regenerated plants, according to the results of qRT-PCR. The content of tanshinone IIA and cryptotanshinone were dramatically enhanced in transgenic regenerated plants and hairy roots by Ultra Performance Liquid Chromatography analysis. Based on the Agrobacterium-mediated transformation of *S. miltiorrhiza*, this study developed a new method for regenerating plants with transgenic hairy roots. This method provides a foundation for the breeding of *S. miltiorrhiza* and the sustainable development of medicinal plant resources, as well as provides a useful reference for the application of other species.

## Introduction

*Salvia miltiorrhiza* Bunge (called Danshen in Chinese) is a perennial herb belonging to Labiatae family (Feng et al., 2019). Its dried roots and rhizomes have been utilized for over 1,000 years in Asia to treat cardiovascular and cerebrovascular ailments (Wu et al., 2012; Jiang et al., 2019). Nowadays, there are a variety of pharmaceutical formulations with Danshen as the major component, which is widely utilized to treat coronary artery disease and acute ischemic stroke, such as Fufang Danshen Dripping Pill and Danshen Injection (Wu et al., 2012; Jiang et al., 2019). In addition, Danshen tea is considered a health food for preventing coronary heart disease and is favored by consumers (Shi et al., 2019).

The active medicinal ingredients of *S. miltiorrhiza* mainly include hydrophilic phenolic acids and lipid-soluble tanshinones. The bioactivities of phenolic acids include antioxidant, anti-inflammatory, anti-cancer, and antibacterial properties (Tyszka-Czochara et al., 2018; Yu et al., 2021). Tanshinones, including dihydrotanshinone I, cryptotanshinone, tanshinone I, and tanshinone IIA, are the major group of bioactive constituents and secondary metabolites of *S. miltiorrhiza*, which possess antitumor, antibiotic, and anti-inflammation activities (Wang et al., 2007; Dong et al., 2011). In clinical practice, tanshinones and chemically modified derivatives are widely used to treat patients with coronary artery disease (Takahashi et al., 2002).

*S. miltiorrhiza* geranylgeranyl diphosphate synthase 1 (*SmGGPPS1*) is the first key enzyme gene in the downstream pathway of tanshinones biosynthesis (Hua et al., 2012). Afterwards, through the catalysis of copalyl diphosphate synthase 1 (CPS1), kaurene synthase-like 1 (KSL1), and multiple Cytochrome P450 enzymes (CYP76AH1, CYP76AH3, CYP76AK1, CYP71D375, and CYP71D373), leading to the formation of a tanshinone skeleton containing the D ring (Gao et al., 2009; Guo et al., 2013, 2016; Ma et al., 2021). Recent reports suggest that oxoglutarate-dependent dioxygenase (2-ODD) superfamily may participate in the synthesis of tanshinone (Xu and Song, 2017; Song et al., 2021). However, the synthesis of tanshinone is *via* a very complicated network, and its downstream synthesis pathway is still unclear. Danshen commercial demand has increased in recent years, while wild resources have decreased. The traditional extraction method from *S. miltiorrhiza* root is no longer sufficient to meet demand. In addition, due to the synthetic pathway of tanshinones has not being clarified, it is difficult to synthesize tanshinones *in vitro*. As a result, some biotechnological approaches must be used to improve tanshinone production. Because of its genetic stability and rapid growth, hairy root culture is a promising strategy for the generation of high-value chemicals in medicinal plants (Shi et al., 2021). To date, the hairy root system has been established in a variety of medicinal plants, including *S. miltiorrhiza*, *Taxus media*, *Panax ginseng*, and *Scutellaria baicalensis* (Hu and Alfermann, 1992; Exposito et al., 2010; Zhang et al., 2015; Zhao et al., 2016). *Agrobacterium rhizogenes* have been shown in studies to transfer key enzyme genes related to secondary metabolism synthesis into recipient plants, effectively increasing the synthesis of target secondary metabolites. Overexpression of *GGPPS1* and *DXS2* in *S. miltiorrhiza* hairy roots, for example, produces higher levels of tanshinone than control lines (Shi et al., 2016).

Many factors affect the transformation efficiency of hairy roots, such as the inconsistent performance of different genotypes of *Agrobacterium* in the same plant (Sharafi et al., 2014). In addition, explant type, bacterial optical density (OD), infection time, co-cultivation time, and antibiotic concentration all have an impact on the *Agrobacterium*-mediated genetic transformation system (Dutta and Kottackal, 2013; Meng et al., 2019). Although hairy root culture in *S. miltiorrhiza* has been established, there have been few studies on the in-depth optimization of its transformation conditions (Hu and Alfermann, 1992; Chen et al., 1999; Kai et al., 2011).

Furthermore, due to the characteristics of hairy roots, only asexual reproduction is possible. To study sexual reproduction, researchers must first induce callus and then harvest complete plants (Wang et al., 2013). Many species, according to research, can produce adventitious shoots or shoots from their roots (Raju et al., 1966). We observed that the hairy roots of *S. miltiorrhiza* can also produce root buds under certain conditions. In this way, the risk of generating mutation is reduced, and the culture time is reduced.

In this study, the transformation efficiency of *Agrobacterium* was improved by optimizing the different transformation factors (OD value, infection time, co-cultivation time, and antibiotic concentration). Then, we devised a simple and quick hairy root culture method for inducing root buds while harvesting transgenic hairy roots, allowing us to obtain transgenic regenerated plants directly. Finally, using this method, the *SmGGPPS1* transgenic hairy root lines and transgenic-positive plants were obtained. We demonstrated the potential of this genetic transformation method in promoting the secondary metabolism of medicinal plants and provided a reference for other species by using qRT-PCR and Ultra Performance Liquid Chromatography (UPLC) to determine the expression of the target gene and the content of active components.

## Materials and Methods

### Plant Materials and Culture Conditions

The white flower *S. miltiorrhiza*, line BH-18, was grown in an experimental area at Shandong Agriculture University in Tai’an (N36.17, E117.17), Shandong Province, China. The mature seeds were surface sterilized by 75% ethanol for 5 min and 0.1% HgCl_2_ solution for 10 min. Subsequently, the seeds were washed 5 times with distilled water and inoculated on MS medium (Murashige and Skoog, 1962). One-month-old plant leaves were collected as explants for transformation. All the controlled environment cultures were incubated in a growth incubator with a 16/8 h light/dark cycle using cool white fluorescent illumination (3,000 lx) and 70% relative humidity at 25°C unless specified.

### Hygromycin Sensitivity Assay

Hygromycin sensitivity experiments were performed to determine the optimal threshold concentration of hygromycin for efficient selection of transformed hairy roots. The leaf sections of size 0.5 cm × 0.5 cm were cultured on MS medium supplied with different concentrations (2.5, 5, 7.5, or 10 mg/l) of hygromycin for 25 days. Leaf explants grown on MS medium without hygromycin were kept as control. After 25 days, calculate the percentage of survival of explants of *S. miltiorrhiza*.

### *Agrobacterium rhizogenes* Strain and Vector Construct Used

**Figure 1:**
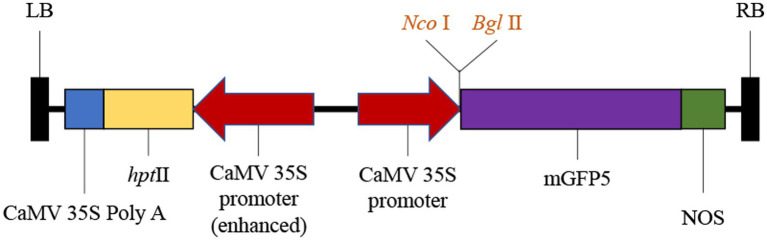
Schematic representation of T-DNA region of binary vector pCAMBIA1302 having restriction sites. LB, left border; *hpt*II, hygromycin B resistance gene; GFP, Green fluorescent protein; *NOS*, nopaline synthase poly A gene terminator; RB, right border.

The open reading frame (ORF) sequence encoding a 1,092-bp nucleotide DNA fragment of *SmGGPPS1* was inserted in vector pCAMBIA1302 (CAMBIA, Australia)^1^ after digestion with *Nco*I and *Bgl*II restriction enzymes. Using the freeze–thaw procedure (Holsters et al., 1978), the plasmid was delivered into the competent *A. rhizogenes* strain *Ar.qual*. In this recombinant vector, the selective marker gene hygromycin phosphotransferase (*hpt*II) and the reporter gene mGFP5 with nopaline synthase (nos) as a terminator were employed; both the reporter and marker genes were driven by distinct CaMV 35S promoters (Figure 1).

### The Optimization of Optical Density (OD), Infection Time, and Co-cultivation Time

The optimization of bacterial OD, infection time, and co-cultivation time was evaluated to achieve the largest transformation frequency in *S. miltiorrhiza*. *A. rhizogenes* strain *Ar.qual* harboring the vector, pCAMBIA1302, was cultivated in TY liquid medium (tryptone 5 g/l, yeast extract 3 g/l, 10 ml 1 M CaCl_2_ solution, pH 7.0) with 50 mg/l streptomycin and 50 mg/l kanamycin at 28°C with shaking (200 rpm). When the OD_600_ reached 0.2,0.4, 0.6, 0.8, or 1.0, the bacterial cells were centrifuged (6,000 rpm, 10 min), and the resultant pellet was resuspended in an equivalent amount of MS media (agar free). To determine the optimal infection time, the leaves (0.5 cm × 0.5 cm) of *S. miltiorrhiza* were treated with *Ar.qual* harboring the pCAMBIA1302 (OD_600_ = 0.6) for 5, 10, 15, 20, or 30 min, then recorded the data. After the optimal infection time wad determined, the cultures were co-cultivated for 1, 2, 3, 4, or 5 days on MS medium at 25°C in dark.

Following co-cultivation, the explants were rinsed five times with 400 mg/l timetin water and blot-dried on sterile filter paper before being transferred to selection medium (MS media supplemented with 7.5 mg/l hygromycin and 400 mg/l timetin) for screening of probable transformants. After 3 weeks, the sub-cultured weekly hairy roots were examined and photographed under UV light (365 nm) by a UV light source (LUYOR-3415RG). The *A. rhizogenes* infection efficiency was estimated by the rate of hairy roots production and GFP expression in *S. miltiorrhiza* explants. The rate of hairy roots production (RHRP, %) = (The number of hairy roots with lengths ≥1 cm/the number of infected explants) × 100%. The rate of GFP expression (RGE, %) = (The number of hairy roots with lengths expressing GFP ≥ 1 cm/total number of hairy roots with lengths ≥1 cm) × 100%. 30 explants were employed for each variable in the above experiment and repeating three times.

### Induction of Radical Buds in Positive Transgenic Hairy Roots and Plant Regeneration

The hairy roots expressing GFP and length ≥ 2 cm were cut and cultivated individually on selection medium for 2 weeks. Afterward, the hairy roots were transferred to 200 ml 6, 7-V liquid medium (supplement 2 mg/l 6-Benzylaminopurine) at 25°C, 110 rpm under 1/23 h light/dark (Veliky and Martin, 1970). After 3 weeks, estimating the number of root buds and the weight of hairy roots after drying. The root buds with partially hairy roots were then transferred on to rooting medium (½ MS medium supplemented with 0.5 mg/l NAA). After 4 weeks, the regenerated plants were tested for GFP and photographed. The plantlets with well-developed roots and GFP expressing were potted in the soil till maturity.

### Molecular Analysis

The DNA was extracted from hairy roots and positive regeneration plant leaves according to a modified CTAB method (Song et al., 2010). Gene-specific primers (hyg-F and hyg-R) were designed by Primer Premier 6.0 (Supplementary Table S1). The PCR condition was set according to Su et al. (2021). The products were photographed using the gel imaging system after electrophoresis on a 1% agarose gel.

Total RNA was isolated from roots of transformed *S. miltiorrhiza* regeneration plants by the Flying Shark™ Universal Plant RNA Kit (Nobelab #RNE35, China). The RNA concentration determination and cDNA synthesis has been described in previous work (Su et al., 2021). All primers used are listed in Supplementary Table S1. qRT-PCR amplification was performed on the Applied Biosystems Q6 System (United States) by a ChamQ™ Universal SYBR qPCR Master Mix kit (Vazyme, China). qRT-PCR reaction condition was performed as reported by Su et al. (2021).

### Quantitative UPLC Analysis of Tanshinones

The 8-week-old transgenic regenerated plant roots and hairy roots (100 mg) were dried and ground into powder, then extracted for 30 min with 5 ml of 80% methanol under sonication (25°C, 40 kHz). After, filtered through a 0.22 μm microfiltration membrane (Fang et al., 2012). Analysis was performed on the ACQUITY UPLC system (Waters Corporation, MA, United States) and an ACQUITY^™^ BEH C18 column (3.0 mm × 150 mm, 1.7 μm; Waters, United States) was used. We selected the gradient elution approach by a mobile phase of acetonitrile (A)—water containing 0.05% phosphoric acid (B): 20% A (0–5 min), 20–30% A (5–10 min), 30–60% A (10–15 min), 60–70% A (15–20 min), 70–80% A (20–25 min), 80–100% A (25–30 min). The injection volume was set at 4 μl, and the flow rate was 0.5 ml/min. The monitoring wavelength was 280 nm. The standard curves of cryptotanshinone and tanshinone IIA referred to the previous work of our group (Mei et al., 2021). The contents were calculated separately using standard curves based on the corresponding peak areas in UPLC.

### Data Analysis

Each experiment was performed in triplicate and data were analyzed using ANOVA to detect the significant differences between means. All data were analyzed with SPSS 24.0 (SPSS Inc., United States) and the difference contrasted using Duncan’s multiple range test and compared at a *p* < 0.05 probability level.

## Results

### Hygromycin Sensitivity Test

In our study, the explants of *S. miltiorrhiza* inoculated on MS medium without hygromycin can survive and induce callus after 25 days. The rate of explants survival and callus induction decreased drastically with the addition of hygromycin to the MS medium. When the concentration of hygromycin reaches 5 mg/l, the survival of explants rate and callus induction rate were only 69.33 and 32% after growing in MS medium for 25 days, respectively (Table 1). At that time, with the growth of most explants stagnated, the chlorophyll pigment begins to decrease. Increased concentration of hygromycin to 7.5 mg/l in MS medium, almost all the explants became necrotic and discolored. On this premise, we chose 7.5 mg/l hygromycin for transformation tests in order to exclude non-transformed explants while allowing converted explants to continue to grow on the MS medium.

**Table 1:** Details of sensitivity test for hygromycin on MS medium.

Concentration of hygromycin (mg/L)	Number of explants	Rate of explants survived (%)	Rate of callus induction (%)
0	25	100^a^	98.67 ± 1.33^a^
2.5	25	98.67 ± 1.33^a^	90.67 ± 2.67^a^
5	25	69.33 ± 4.81^ab^	32.00 ± 4.00^b^
7.5	25	2.67 ± 1.33^b^	0
10	25	0	0

The experiment was repeated three times, and the results were expressed as mean ± standard error. For each concentration of hygromycin, means with the same letter are not significantly different at 0.05 level according to Tamhane’s T2 multiple range test. Rate of explants survived (%) = (The number of survival explants/all the number of explants) × 100%. Rate of callus induction (%) = (The number of callus/all the number of explants) × 100%.

### Optimization of *Salvia miltiorrhiza* Genetic Transformation System Mediated by *Agrobacterium rhizogenes*

The leaves of *S. miltiorrhiza* were introduced as explants for *Agrobacterium*-mediated transformation studies. In the present study, three factors affecting transformation efficiency were researched separately, including bacterial optical density, infection time, and co-cultivation time.

#### Effect of Optical Density of *Agrobacterium rhizogenes* on *Salvia miltiorrhiza* Transformation

**Figure 2:**
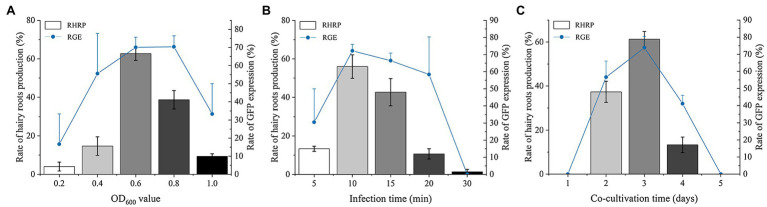
Factors affecting the transformation efficiency of *S. miltiorrhiza* explants. **(A)** Effect of optical density on RHRP and RGE at 0.2, 0.4, 0.6, 0.8, and 1.0 OD_600_. **(B)** Effects of infection time on RHRP and RGE for 5, 10, 15, 20, and 30 min. **(C)** Effects of co-cultivation time on RHRP and RGE for 1, 2, 3, 4, and 5 d. Results are expressed as mean ± standard error. RHRP (%) = (The number of hairy roots with lengths ≥1 cm/the number of infected explants) × 100%. RGE (%) = (The number of hairy roots with lengths expressing GFP ≥ 1 cm/total number of hairy roots with lengths ≥1 cm) × 100%.

The cultures of 0.2, 0.4, 0.6, 0.8, and 1.0 OD_600_ were used to infect the explants of *S. miltiorrhiza* to obtain optimal transformation efficiency. With the increase of OD_600_, the RHRP (the rate of hairy roots production) and RGE (the rate of GFP expression) continued to increase (Figure 2A). When the OD_600_ increased to 0.6 and 0.8, the RGE reached a very high level, 70.08 and 70.37%, respectively. Therefore, the highest transformation efficiency was discovered for OD_600_ ranging from 0.6 to 0.8. However, the RHRP was only 38.67% at OD_600_ = 0.8, while 0.6 OD_600_ can reach 62.67%. Thereafter, further increase of OD_600_ up to 1.0 resulted in a decline in the RHRP and RGE, and the explants became brownish. These data suggest that there are considerable disparities in transformation efficiency across various OD treatments. In this experiment, the optimal OD600 is 0.6.

#### Effect of Infection Time on *Salvia miltiorrhiza* Transformation

Five different infection times (5, 10, 15, 20, and 30 min) were assessed to investigate the influence of infection on *S. miltiorrhiza* transformation efficiency. The infected explants exhibited a high RGE (72.21%) by the best concentration of *Ar.qual* for 10 min. Notably, both the RHRP and GRE decreased after co-cultivation with *Ar.qual* as the infection time increased over 10 min (Figure 2B). These results showed that appropriate infection time can improve the genetic transformation efficiency of *S. miltiorrhiza*.

#### Effect of Co-cultivation Time on *Salvia miltiorrhiza* Transformation

To verify the effect of co-cultivation time on *S. miltiorrhiza* transformation, five cultivation time groups (1, 2, 3, 4, and 5 days) of infected explants on MS medium were assessed. The results revealed that no hairy roots were produced for 1 day and 5 days of co-cultivation (Figure 2C). When co-cultured for 3 days, the RHRP and RGE reached the highest, which were 61.33 and 73.85%, respectively.

### Induction of Root Buds in Positive Transgenic Hairy Roots and Plantlet Regeneration

**Figure 3:**
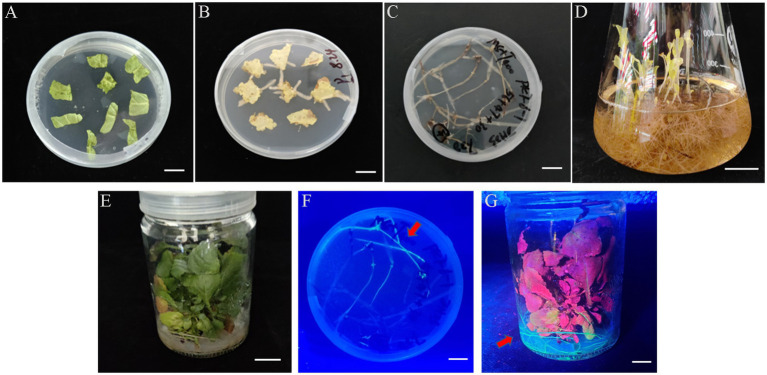
The experimental process of *Agrobacterium*-mediated transformation in *S. miltiorrhiza*. **(A)** Explants. **(B)** Selected culture. **(C)** Hairy roots. **(D)** Root bud appearance. **(E)** Plant regeneration. **(F)** Stable GFP expression in transgenic hairy roots. **(G)** Stable GFP expression in transgenic plant. Bars = 1 cm.

We determined that the *Ar.qual* was collected at OD_600_ = 0.6, infecting explants for 10 min, and co-cultured for 3 days as the optimal condition for genetic transformation of *S. miltiorrhiza*. Afterward, the explants were filtered on a selection medium (containing 7.5 mg/l hygromycin), and the selected hairy roots were transferred to a 6, 7-V liquid medium for expansion culture. Here we found an interesting phenomenon, adding 2 mg/l 6-BA to the liquid medium and applying light to the hairy roots for 1 h per day can induce the hairy roots to produce a member of radical buds. However, the yield of hairy roots did not perform a dramatic change compared with the control (Supplementary Table S2). Subsequently, we transferred the radical buds to the rooting medium. After 30 days, new roots were taken below the radical buds to complete plantlet regeneration (Figures 3A–E). After GFP detection, a total of 9 positive transgenic hairy root lines and 11 positive transgenic regenerated plants were obtained (Figures 3F,G).

### Molecular Analysis of the Positive Transgenic *Salvia miltiorrhiza* Lines

**Figure 4:**
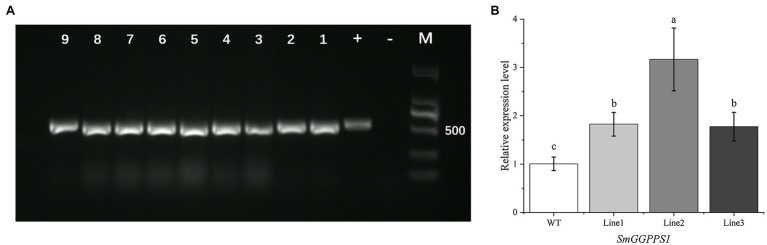
**(A)** PCR detection of *hpt*II gene in transgenic plants, with pCAMBIA1302 vector as a positive control (+) and wild-type plants as a negative control (−), transgenic hairy root lines (1–4), transgenic regenerated plant leaves (5–9). **(B)** Relative expression level of *SmGGPPS1* in overexpression transgenic regenerated plants by qRT-PCR analysis. Actin gene in *S. miltiorrhiza* was used as a reference for normalization. WT, wild-type plants; Line 1, 2, 3: roots of transgenic regenerated plants. Error bars represent means ± SD (*n* = 3). Different letters indicate significant differences (*p* < 0.05). The multiple comparison method used for the average level in this study was Duncan.

Genomic DNA from nine transgenic lines randomly selected for PCR analysis showed the expected 531 bp band for the *hpt*II gene (Figure 4A). A positive control was the plasmid of the pCAMBIA1302 vector, while a negative control was wild-type plants. This result reconfirmed that the target plasmid has been successfully transferred into the test sample, and the positive rate of plants through root bud regeneration reached 100%.

For further analysis, qRT-PCR was performed to investigate the level of *SmGGPPS1* expression in the transgenic regeneration plant roots. The histogram clearly illustrated that the expression level of the *SmGGPPS1* in the tested transgenic lines increased 1.82-, 3.17-, and 1.77-fold higher than that in the wild-type (Figure 4B). This indicated that *SmGGPPS1* was successfully overexpressed in positive transgenic regenerated plants.

### Tanshinones Content in Transgenic Regenerated Plants and Hairy Roots of *Salvia miltiorrhiza*

**Figure 5:**
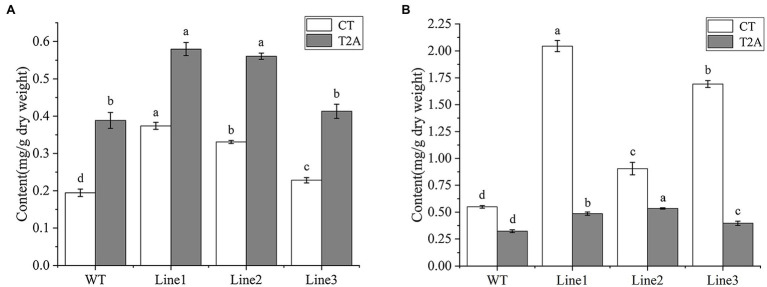
UPLC analysis of cryptotanshinone and tanshinone IIA in transgenic root lines. **(A)** Transgenic regenerated plant root lines. **(B)** transgenic hairy root lines. WT, the root of wild-type plants; CT, cryptotanshinone; T2A, tanshinone IIA. Error bars represent means ± SD (*n* = 3). Different letters indicate significant differences (*p* < 0.05). The multiple comparison method used for the average level in this study was Duncan.

To determine the cryptotanshinone (CT) and tanshinone IIA (T2A) production, UPLC analysis was performed on the roots of transgenic regenerated plant (RTRP) and transgenic hairy roots (THR). The retention time of CT and T2A were approximately 19.6 and 22.3 min, respectively (Supplementary Figure S1). All the transgenic lines of the RTRP and THR showed a higher level of CT and T2A than WT. Of these, the highest CT and T2A production was detected in line 1 of the RTRP with the concentration of 0.37 and 0.58 mg/g, respectively (Figure 5A). In the THR, the highest concentration of CT and T2A were 2.04 and 0.53 mg/g, respectively (Figure 5B). This result confirmed that the RTRP and THR with high tanshinones content can be rapidly obtained by our transformation and culture method, and it once again verified that *SmGGPPS1* is one of the key enzyme genes for tanshinones synthesis (Kai et al., 2011; Hua et al., 2012).

## Discussion

The biosynthesis of plant secondary metabolites can be affected by environmental conditions and different stages of plant development. Many medicinally valuable secondary metabolites tend to be found in very low levels in plants. For example, taxol is a natural secondary metabolite isolated and purified from the bark of *Taxus chinensis*, which has significant antitumor effects. However, the content in plant tissue was quite low, only contain 0.01% (Croteau et al., 2006). The bioactive component icaritin in *Epimedium* is less than 2% of dry plant matter (Pw et al., 2021). Currently, secondary metabolite production *via* hairy roots is regarded as an efficient and feasible method. The explants are infected with the *A. rhizogenes* Ri plasmid, which can rapidly produce a large number of hairy roots and has higher levels of bioactive metabolites than wild plants (Sevón and Oksman-Caldentey, 2002; Shi et al., 2021).

The genetic transformation of *S. miltiorrhiza* hairy roots has been gradually established and optimized over the past 30 years (Hu and Alfermann, 1992; Chen et al., 1999; Kai et al., 2011). In previous studies, the *A. rhizogenes* strains ATCC15834, C58C1 (pRiA4), and ACCC10060 were used to induce hairy roots of *S. miltiorrhiza* (Chen et al., 2001; Kai et al., 2011; Xu and Song, 2017). However, *A. rhizogenes* may exhibit different infectivity abilities due to the different susceptibility of the strains to the host (Yang and Yang, 2009). In this study, the successful application of *Ar.qual* strain in *S. miltiorrhiza* was reported for the first time, with the hairy root production rate reaching 62.67%. Furthermore, we tried to improve the transformation efficiency of *S. miltiorrhiza* by optimizing the OD value, infection time, and co-cultivation time. When the concentration of *A. rhizogenes* was too high to clear it from the explants, which will eventually led to *Agrobacterium* contamination. However, if the concentration of *A. rhizogenes* is too low, the infection ability is weak, and the production of hairy roots is seriously reduced (Satish et al., 2017). In this paper, we discovered that OD_600_ = 0.6 is the optimal concentration for infection, which is consistent with the findings of other transformation systems (Li et al., 2017; Hu et al., 2020). Furthermore, infection time and co-cultivation time had a significant impact on *S. miltiorrhiza* transformation efficiency. To obtain the highest hairy root yield and GFP expression rate, the explants were infected with OD_600_ = 0.6 for 10 min and co-cultured for 3 days. Longer infection and co-culture times may result in bacterial overgrowth, allergic necrosis in explants, and, ultimately, a reduction in transformation efficiency (Ombori et al., 2013).

Hygromycin has been widely used in plant genetic transformation as a selective agent that effectively kills eukaryotic cells by inhibiting protein synthesis (Yu et al., 2016). In our study, the hygromycin concentration was gradually increased from 2.5 to 10 mg/l. Low concentrations of hygromycin (2.5 mg/l) had no effect on the growth of *S. miltiorrhiza* explants, according to the results. Following that, high concentrations of hygromycin (7.5 mg/l) disrupted the growth and proliferation of untransformed cells, significantly lowering the false positive rate. *S. miltiorrhiza* was found to be more sensitive to hygromycin than previously reported crops, such as *Spathiphyllum cannifolium* and finger millet (Yu et al., 2016; Satish et al., 2017).

Although hairy roots grow rapidly, no secondary metabolites have been industrially produced through hairy roots, due to insufficient yield and high cost (Shi et al., 2021). Furthermore, hairy roots typically grow underground and are unable to differentiate into stems, leaves, flowers, and other organs and tissues, making phenotype observation and self-breeding research difficult. As a result, it is critical to obtain transgenic regenerated plants as quickly as possible. Previous research has shown that when transgenic-positive hairy roots were transferred to a medium containing 2, 4-D and 6-BA, the hairy roots could be induced to regenerate embryogenic callus after a period of culture (Wang et al., 2013). Following that, transfer to a sprout medium allows the callus to regenerate shoots, which requires a series of subcultures that are time-consuming and labor-intensive, as well as increasing the risk of mutations (Arockiasamy and Ignacimuthu, 2007).

**Figure 6:**
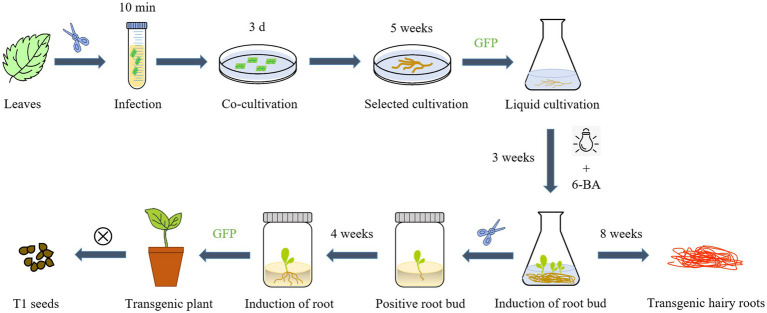
An overview of the main steps for *A. rhizogenes* transformation and one-step regeneration of *S. miltiorrhiza*.

*S. miltiorrhiza* is a perennial herb that, like many herbs and woody plants, produces adventitious shoots from the root. Previous studies have found that the hairy roots of the perennial herbs *Hyoscyamus muticus* and *Plumbago indica* can grow root buds directly after a period of liquid culture (Kirsi-Marja et al., 1991; Gangopadhyay et al., 2010). We discovered that under certain light and hormone conditions, the hairy roots of *S. miltiorrhiza* can directly produce root shoots. As a result of taking advantage of this feature of *S. miltiorrhiza*, we developed a new process for the hairy roots culture of *S. miltiorrhiza*. Using *A. rhizogenes* genetic transformation, positive hairy roots were induced to directly produce a large number of root buds by adding 6-Benzylaminopurine (2 mg/l) to the 6, 7-V liquid medium for culturing hairy roots and performing short-term light exposure (1 h/d). This method can save time and reduce the danger of media contamination by skipping the callus-mediated regeneration step. After that, positive root buds were identified by GFP detection and transferred to rooting medium, whereas hairy roots were cultivated in a 6, 7-V liquid medium. Hairy roots and transgenic regenerated plants with high tanshinone content can be obtained after 8 weeks (Figure 6).

Previous research has demonstrated that improving *Agrobacterium tumefaciens* transformation conditions can increase the positive rate of transgenic regenerated *S. miltiorrhiza* plants to 70% (Yan and Wang, 2007). GUS staining was performed on the leaves of the regenerated plants in his investigation to see if the transgene was successful. Our technique, on the other hand, can screen positive hairy roots in real time using GFP detection at an early stage. The transgenic plasmid was found in 100% of the regenerated plants from root buds triggered by positive hairy roots, according to PCR data. This suggests that using GFP detection to boost the positive rate of regenerated plants in this method is a good idea. *SmGGPPS1* was successfully overexpressed in transgenic regenerated plants, and the concentration of CT and T2A in the roots of transgenic regenerated plants was much higher than in WT, according to qRT-PCR and UPLC analyses. Simultaneously, we discovered that CT content was higher than T2A in the transgenic hairy root, which was consistent with previous research (Kai et al., 2011; Wei et al., 2019). Recent studies have shown that *SmTIIAS* can convert CT to T2A (Song et al., 2021), thus we hypothesize that more CT may be accumulated in hairy roots due to the influence of the environment in liquid medium on the expression of *SmTIIAS*.

## Conclusion

This study optimized the transgenic parameters of *S. miltiorrhiza* mediated by *A. rhizogenes* and developed a one-step regeneration method for *S. miltiorrhiza* hairy roots, allowing for the rapid production of transgenic *S. miltiorrhiza* plants and hairy roots with high tanshinone content. This method not only reduces labor intensity but also saves a significant amount of time and material cost. This efficient protocol has the potential to be used in secondary metabolism and self-breeding studies of *S. miltiorrhiza*. At the same time, it would be a useful reference for other plant species’ transformation research.

## Data Availability Statement

The original contributions presented in the study are included in the article/Supplementary Material, and further inquiries can be directed to the corresponding author.

## Author Contributions

YS, CL, and JW conceived and designed the experiments and wrote and revised the manuscript. YS, CL, JZ, BH, and JW performed the experiments. YS, JL, and SW analyzed the data. YS, RL, and XL generated the pictures. ZS modified the language. All authors read and approved the final manuscript.

## Funding

This work was financially supported by Shandong Modern Agricultural Industry Technical System Project of China (SDAIT-20-04), Shandong Province Key Research and Development Plan of China (2017CXGC1302), National Key Research and Development Program of China (2017YFC1702705), National Natural Science Foundation of China (81872949), and Natural Science Foundation of Shandong province of China (ZR2019HM081).

## Conflict of Interest

The authors declare that the research was conducted in the absence of any commercial or financial relationships that could be construed as a potential conflict of interest.

## Publisher’s Note

All claims expressed in this article are solely those of the authors and do not necessarily represent those of their affiliated organizations, or those of the publisher, the editors and the reviewers. Any product that may be evaluated in this article, or claim that may be made by its manufacturer, is not guaranteed or endorsed by the publisher.

## Footnotes

^1^ http://www.cambia.org
