# Supplementary material for: One-Step Regeneration of Hairy Roots to Induce High Tanshinone Plants in Salvia miltiorrhiza
Source: Front Plant Sci. 2022 May 20;13:913985. doi: 10.3389/fpls.2022.913985 (PMC9163987; doi:10.3389/fpls.2022.913985)
Supplement: Supplementary file 1 [file Table_1.DOCX]

Supplementary Material

**Table S1︱Primer sequences for PCR and quantitative PCR**

| **Primer names** | **Primer sequences (5'-3')** | **Product sizes (bp)** |
| --- | --- | --- |
| hyg-F | ATGGTTTCTACAAAGATCGTTATGT | 531 |
| hyg-R | TGTTGGCGACCTCGTATTGG |  |
| qPCR-Actin-F | GGTGCCCTGAGGTCCTGTT | 267 |
| qPCR-Actin-R | AGGAACCACCGATCCAGACA |  |
| qPCR-GGPPS-F | GGGGCTATTTTGGGAGGTGGAA | 184 |
| qPCR-GGPPS-R | CAGCAGCTTGGGATACGTGGTC |  |

**Table S2︱Effects of light and 6-BA on induced root buds**

| **medium** | **Light** | **6-BA** | **No. of root buds** | **Hairy root dry weight (g)** |
| --- | --- | --- | --- | --- |
| 6,7-V | - | - | 0 | 3.04 ± 0.31^a^ |
| 6,7-V | 1h/d | - | 6.67 ± 0.88^a^ | 2.56 ± 0.30^a^ |
| 6,7-V | - | 2.0 mg/L | 0.67 ± 0.33^b^ | 2.89 ± 0.19^a^ |
| 6,7-V | 1h/d | 2.0 mg/L | 10.33 ± 1.76^a^ | 2.64 ± 0.26^a^ |

The experiment was repeated three times, and the results were expressed as mean ± SE. Means followed by the same letters within a column do not differ significantly at p ≤ 0.05 according to Duncan’s multiple range test.


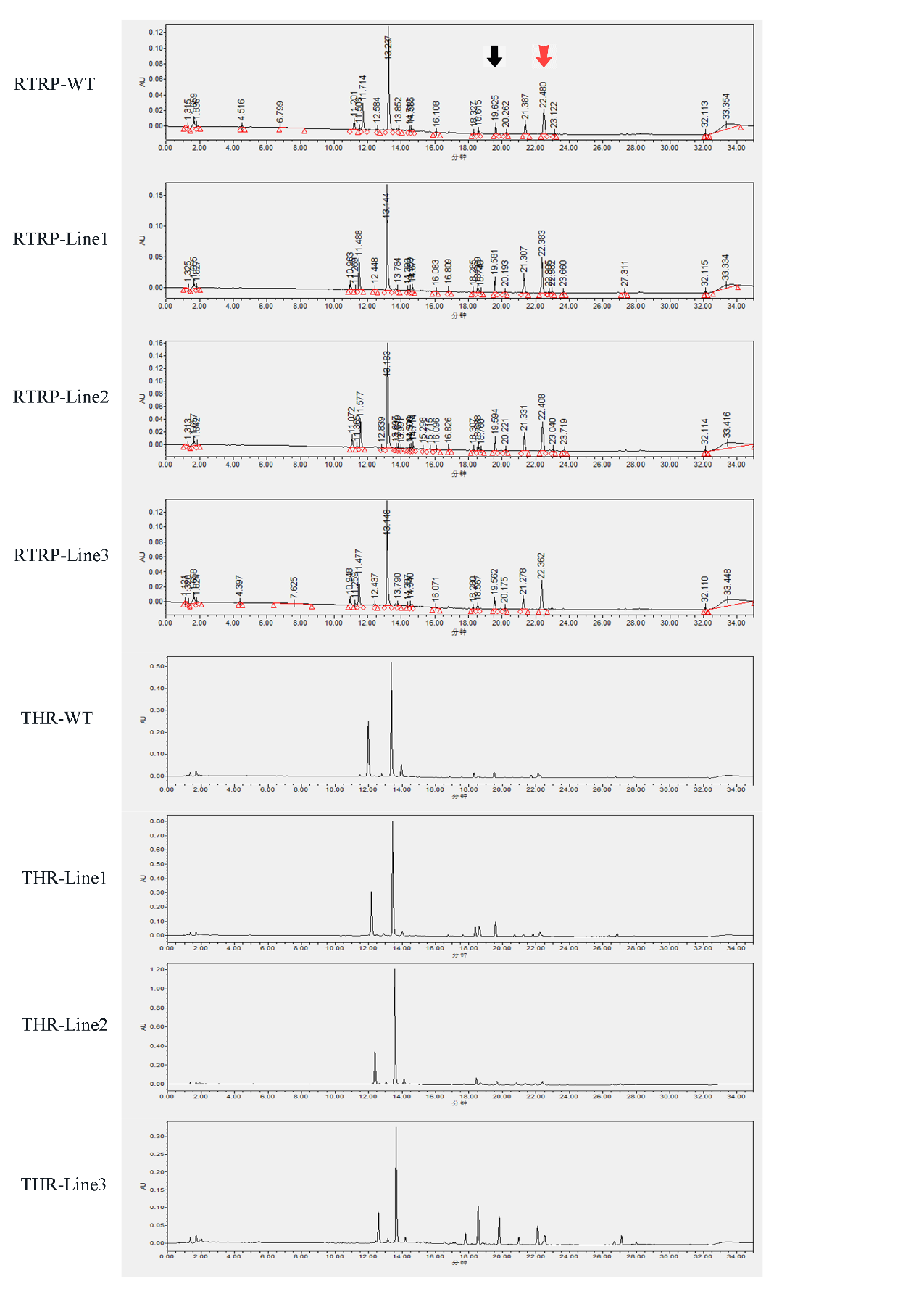


**Fig. S1 UPLC metabolite profiles of tanshinones in transgenic *S. miltiorrhiza* plants and hairy roots lines.** Black arrow: Cryptotanshinone; Red arrow: Tanshinone IIA; RTPR: The overexpressed *SmGGPPS1* transgenic regenerated plants; THR: The overexpressed *SmGGPPS1* hairy root lines.
